# Lunacy Reform.—II
*The first article appeared in our July issue.


**Published:** 1922-08

**Authors:** H. J. Norman

**Affiliations:** (Lecturer on Mental Diseases, Westminster Hospital, Senior Assistant Medical Officer at Camberwell House)


### LUNACY REFORM.*—II.

**By H. J. NORMAN, M.B., Ch.B. (Lecturer on Mental Diseases, Westminster Hospital, Senior Assistant Medical Officer at Camberwell House).**

IT is interesting to note—apropos the remarks anent the early treatment of patients suffering from mental disorders in the previous article—that a Government Bill is to be introduced at an early date to permit this without certification. According to "The Times," a sufficient measure of agreement was reached at a conference between the Minister of Health, the Lunacy Commissioners and medical men who are Members of Parliament to ensure a Bill being drafted immediately. Its effect will be to permit medical men, for a period of six months, to treat the mentally deranged, in appropriate cases, without certifying them as lunatics.† The writer in "The Times" is unduly optimistic when, commenting on the fact that about 50,000 persons are now certified annually, he says that "half the number, it is estimated, ought never to be sent to asylums"! It is to be hoped that this Bill will pass successfully and speedily into law, and that it will provide, as already suggested, for adequate inspection of the homes where the treatment is to be carried out. The probationary period should be allowed also to patients who will have to go to asylums or licensed houses on account of the acuteness of their symptoms precluding them from being nursed in private houses. If this is not done a hardship will be inflicted on patients, often quite young, who suffer from attacks of acute mania or of confusional insanity and in whom the symptoms which, at present, render certification necessary are transient in character.

In considering reforms in asylum life, due thought should be given to the question of providing not only for the patients, but also for the staff. It is customary at the present time to accept almost any stick wherewith to beat those who have charge of mental patients in asylums! As Dr. Wolseley Lewis remarked at the recent conference, "There still lingers a prejudice in the public mind, which, however ill-founded, is readily awakened by such articles in the Press as those inspired by Dr. Lomax's book." With some people the ideal system would be to enlist the services of those who are supposed to

* The first article appeared in our July issue.

† "The Times," June 22, 1922.

have a vocation for such duties; but in this workaday world—and with economic conditions as they are—it is unlikely that we shall find even a tithe of the necessary number in this way. Dr. Bedford Pierce put the matter concisely—"When one sees the character of the work one cannot but feel that the nurses do not receive the consideration, the pay, or the status that their work for the community deserves." Many suggestions have been made for increasing the efficiency of the mental nurse; and there is a tendency even now to make the burden of examinations unduly heavy—or, perhaps, it would be fairer to say, to set a rather too high standard of theoretical knowledge! By all means let us have the best possible nurses and let them be trained to the highest degree of skill. But it must not be forgotten that, in order to achieve this, it will be necessary to make the profession a desirable one from the nurses' point of view. There are other aspects of the matter which merit discussion, but this may suffice meantime to indicate that the nursing problem alone requires ample consideration in any scheme of lunacy reform.

The comfort and well-being of the staff are important intrinsically, but also have a bearing upon the attitude of the staff to the patients. This must be borne in mind; for it is no use considering such matters from an abstract point of view. If an army "marches on its stomach," it is no less true that good and reliable work—especially if it be of long duration—can be done only by people whose energies are not diverted from their proper purpose. The nursing of mental patients may be—and often is—most trying and arduous, necessitating self-control and forbearance under exacting conditions. It would be well if those who are too apt to think evil of mental nurses would recollect this before passing judgment because of some hasty word or action.

We are often held up to scorn by other nations because of our lack of imagination in the matter of diet. The reproach does sometimes seem to be deserved; in any case, it is one that the reformers use in respect to asylum authorities. Sir Harry Lauder has a good story that seems to point to a similar state of affairs in the case of his landlady, who always suggested chops—*et prœterea nihil.* When he took home some sausages she did not even know how to cook those, so he told her to do them like fish. In due course she served up the skins. When he remonstrated, she said she had done as he told her—and cleaned them first! Things are not as bad as that, as a rule, but we are creatures of habit—for the most part it is as well we are, or the daily routine would be infinite labour—and even cooks tend to wearisome repetitions. The result is that in asylums—as elsewhere—there may be an absence of variety in regard to food. The blame is not, of course, to be apportioned solely to the cook. The economic question has to be answered here also. But, allowing for that, every effort should be made to provide as many changes as possible, especially for those patients who are able to appreciate them. For many of the "chronic" patients variety counts far less than quantity. This æsthetic point of view will—as far as one can see at present—always be more important to the appreciative individual than the other way of looking at the question, namely, reckoning the number of calories in an adequate diet. Naturally, the nutritive value of the constituents must not be lost sight of; but it is not a bad rule to go by—to see that the patient gains, or does not lose, weight, and is generally in good physical condition.

It was Lady Mary Wortley Montague who said, in her summary of Lord Lyttleton's advice, that we should "be plain in dress, and sober in our diet," but that plainness and sobriety would, we may be sure, have been liberally interpreted by both of them! Carlyle has insisted on the necessity of not allowing clothes to obscure for us the man within them; "but," he says, "the suit of clothes pretending that it is both clothes and man——!" There is the other side of the matter. Not only are we judged by the clothes we wear, but there is the undoubted influence which our clothing has upon us, psychologically as well as physiologically. This being so, it is necessary to consider clothing as an adjunct to treatment. For those patients who are able to appreciate the importance of well-fitting clothing every effort should be made to let them retain—or reacquire—the sense of individuality which can be ministered to in this way. This is, of course, the rule in private institutions and also, to some extent, in public ones; but it is still too often the case that, as Dr. Rotheram pointed out, the clothing supplied in county and borough mental hospitals, "though generally of excellent quality, is too institutional in character, of too old-fashioned cut and insufficiently well fitted." He went on to say that in a few institutions it has been found possible to permit patients to wear their own private clothing, when friends are willing and able to supply it. When this cannot be done, "each patient, except, perhaps, those who are of dirty and destructive habits, should be supplied with his own outfit of clothes."

Many other matters were dealt with by the conference—research and pathological work, the medical staff, and so on. It may perhaps be possible to deal with some of these later. In the meantime enough has been said to demonstrate that a lively interest is being taken in the numerous questions which arise in connection with the amelioration of asylum conditions.

### THE CENTRAL COUNCIL FOR INFANT AND CHILD WELFARE.

In its report for the year ended March 31, 1922, the Central Council for Infant and Child Welfare makes grateful acknowledgment of the generosity of the Carnegie Trustees, who provided the new offices at Carnegie House, 117 Piccadilly, W.1. Here not only the Central Council, but seven of its constituent bodies, have excellent rooms. The Joint Council of the Order of St. John and British Red Cross Society, by agreement with the Carnegie Trustees, has undertaken to pay £2,000 a year towards the rates, taxes and upkeep of the house. Since February, 1921, County Federations have been formed for Yorkshire, Durham, Cheshire, Northumberland, and the North-West (Lancashire, Westmorland and Cumberland), and it is hoped that others will be added this year. In January last the Council decided to purchase the Child Welfare Travelling Exhibition of the National Council of Women, which can now be hired by local authorities and voluntary societies.

